# LDL-cholesterol-lowering effect of plant sterols and stanols across different dose ranges: a meta-analysis of randomised controlled studies

**DOI:** 10.1017/S0007114514000750

**Published:** 2014-04-30

**Authors:** Rouyanne T. Ras, Johanna M. Geleijnse, Elke A. Trautwein

**Affiliations:** 1 Unilever R&D Vlaardingen, Olivier van Noortlaan 120, PO Box 114, 3130 ACVlaardingen, The Netherlands; 2 Division of Human Nutrition, Wageningen University, Bomenweg 2, 6703 HDWageningen, The Netherlands

**Keywords:** Plant sterols, Plant stanols, LDL-cholesterol, Dose–response methods, Meta-analyses

## Abstract

Phytosterols (PS, comprising plant sterols and plant stanols) have been proven to lower LDL-cholesterol concentrations. The dose–response relationship for this effect has been evaluated in several meta-analyses by calculating averages for different dose ranges or by applying continuous dose–response functions. Both approaches have advantages and disadvantages. So far, the calculation of averages for different dose ranges has not been done for plant sterols and stanols separately. The objective of the present meta-analysis was to investigate the combined and separate effects of plant sterols and stanols when classified into different dose ranges. Studies were searched and selected based on predefined criteria. Relevant data were extracted. Average LDL-cholesterol effects were calculated when studies were categorised by dose, according to random-effects models while using the variance as weighing factor. This was done for plant sterols and stanols combined and separately. In total, 124 studies (201 strata) were included. Plant sterols and stanols were administered in 129 and fifty-nine strata, respectively; the remaining used a mix of both. The average PS dose was 2·1 (range 0·2–9·0) g/d. PS intakes of 0·6–3·3 g/d were found to gradually reduce LDL-cholesterol concentrations by, on average, 6–12 %. When plant sterols and stanols were analysed separately, clear and comparable dose–response relationships were observed. Studies carried out with PS doses exceeding 4 g/d were not pooled, as these were scarce and scattered across a wide range of doses. In conclusion, the LDL-cholesterol-lowering effect of both plant sterols and stanols continues to increase up to intakes of approximately 3 g/d to an average effect of 12 %.

Phytosterols (PS), comprising both plant sterols and plant stanols, are compounds that naturally occur in all foods of plant origin such as vegetable oils, nuts, seeds, grain products, fruits and vegetables. The intake of naturally occurring PS from the general diet is about 200–400 mg/d^(^ ^1^ ^–^ ^3^ ^)^. Higher PS intakes can be achieved by consuming vegetable-based diets such as vegetarian diets for which PS intakes are almost doubled^(^ ^4^ ^,^ ^5^ ^)^ or by consuming food products enriched with PS. PS-enriched foods are well known for their total cholesterol- and especially LDL-cholesterol-lowering properties^(^ ^6^ ^)^. Having elevated LDL-cholesterol concentrations is one of the most important risk factors for CVD. PS-enriched foods are considered a valuable option as part of healthy diet and lifestyle changes in the management of hypercholesterolaemia^(^ ^7^ ^,^ ^8^ ^)^.

Since the 1950 s, abundant research into the LDL-cholesterol-lowering effect of PS has been carried out and this wealth of evidence has been summarised in several meta-analyses^(^ ^6^ ^,^ ^9^ ^–^ ^12^ ^)^. In these meta-analyses, the dose–response relationship for the LDL-cholesterol-lowering efficacy of PS has been investigated. The meta-analyses carried out by Law^(^ ^9^ ^)^, Katan *et al.* ^(^ ^6^ ^)^ and Abumweis *et al.* ^(^ ^10^ ^)^ described a dose–response relationship based on the calculation of average LDL-cholesterol-lowering effects for different categories of PS doses. More recently, Demonty *et al.* ^(^ ^11^ ^)^ have investigated a continuous dose–response relationship, as determined by a first-order elimination function based on the assumption that processes involved in cholesterol transport and absorption are saturable. Musa-Veloso *et al.* ^(^ ^12^ ^)^ subsequently established similar continuous dose–response curves, but this time for plant sterols and stanols separately. Overall, these analyses concluded that with an increasing dose of PS, the LDL-cholesterol-lowering effect increases, but that this effect tapers off at doses of 2–3 g/d.

The applied approaches used to study the dose–response relationship differ between showing average effects for ranges of doses and establishing continuous dose–response functions. Both approaches have advantages and disadvantages. Establishing a continuous dose–response relationship has the advantage that it allows predicting effects for a given dose of PS. However, the shape of the curve largely depends on the distribution of studies across the entire range of doses; if this distribution is not balanced, this type of analysis may become vulnerable for over- or underestimation of the estimated effects at certain doses. For example, in the meta-analysis carried out by Musa-Veloso *et al.* ^(^ ^12^ ^)^, the depicted plant sterol curve clearly underestimated the effects of plant sterols at doses of 2·7–3·3 g/d. As a result, it was suggested that a larger maximal lowering effect exists for plant stanols than for plant sterols. The calculation of average effects for predefined ranges of PS doses is less sensitive to potential over- or underestimation, but this approach does not allow predicting effects over a continuous range of doses.

So far, the calculation of weighed averages for different dose ranges has not been done for plant sterols and stanols separately. Such an analysis would provide useful insights into the comparison of the LDL-cholesterol-lowering efficacy of these two types of PS for which some debate exists^(^ ^12^ ^–^ ^15^ ^)^. Therefore, the main objective of the present analysis was to investigate the combined and separate LDL-cholesterol-lowering effects of plant sterols and stanols when classified into different dose ranges. It was hypothesised that plant sterols and stanols would exert a similar LDL-cholesterol-lowering effect at least up to intakes of, on average, 3 g/d^(^ ^16^ ^)^.

## Experimental methods

### Search strategy and selection of eligible studies

To retrieve potentially relevant human studies eligible for the present analysis, we relied on the systematic searches carried out by the authors of the two most recent meta-analyses^(^ ^11^ ^,^ ^12^ ^)^ that used almost identical search strategies. In the meta-analysis carried out by Demonty *et al.* ^(^ ^11^ ^)^, eighty-one studies with 141 study arms were included, whereas in the more recent meta-analysis carried out by Musa-Veloso *et al.* ^(^ ^12^ ^)^, 114 studies with 182 study arms were included. To retrieve eligible studies that had been published after these two meta-analyses, an additional search was carried out using nine databases (MEDLINE, Embase, BIOSIS, CAB Abstracts, FROSTI, Food Science and Technology Abstracts, Chemical Abstracts, PASCAL and AGRICOLA) from September 2010 to September 2011. Again, identical search terms were used, limited to human studies with no restriction on language.

Based on the criteria described in the two most recent meta-analyses^(^ ^11^ ^,^ ^12^ ^)^, we formulated the following criteria for selecting more recently published studies: (1) randomised controlled studies in human adults; (2) treatment with 4-desmethylsterols and/or 4-desmethylstanols extracted from vegetable oils such as soyabean oil, rapeseed oil and tall oil (so no ferulated PS such as those from rice bran oil or shea nut oil); (3) investigation of blood lipids as primary or secondary outcomes; (4) absence of a co-intervention from which the intake of PS-enriched foods or supplements could not be isolated; (5) availability of relevant LDL-cholesterol data; (6) use of proper placebo in the control group/period; (7) consumption of PS for at least 2 weeks; (8) dose of PS not exceeding 10 g/d; (9) no studies including colectomised patients because it cannot be excluded that colectomy does not have an impact on efficacy.

### Data extraction and statistical analysis

For the present analysis, the following data were extracted: reference information (first author and year of publication); study design (parallel or cross-over); number of subjects (sample size); test product characteristics (dose, type of PS (plant sterols or plant stanols or mix) and food format); the placebo-adjusted relative (%) change in LDL-cholesterol concentration plus accompanying variance measure. In case relative changes were not reported, these were calculated as follows:

For parallel studies, 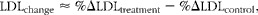
where
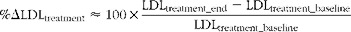
and
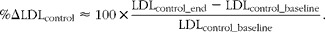



For cross-over studies, 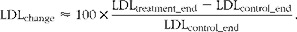



When LDL-cholesterol concentrations were measured at various time points during the intervention, the concentration corresponding to or closest to the 4-week time point was taken for the analysis. When variance measures of the relative changes were not provided and could not be retrieved based on *P* values or 95 % CI, these were calculated using variance measures at baseline and end of the intervention in active and placebo groups/periods assuming, based on an earlier investigation^(^ ^17^ ^)^, a within-subject correlation coefficient of 0·8.

Human intervention studies were divided into six categories based on their PS dose: dose < 1·0 g/d; ≥ 1·0 dose < 1·5 g/d; ≥ 1·5 dose < 2·0 g/d; ≥ 2·0 dose < 2·5 g/d; ≥ 2·5 dose < 3·0 g/d; ≥ 3·0 dose ≤ 4·0 g/d. This approach was chosen so that the incremental dose step was 0·5 g/d except for the lowest and highest categories as the number of studies using doses < 0·5 and between 3·5 and 4·0 g/d was rather limited (*n* 6 each). Study arms with doses exceeding 4 g/d were scarce (*n* 5) and scattered across a wide range of PS doses (5·8–9·0 g/d); therefore, pooling these studies into a single category was judged to be inappropriate; these studies were solely used for descriptive purposes. For each study, the PS dose was determined by the actual dose administered; when not reported, the intended dose was used. Throughout this article, the doses of plant sterols/stanols are expressed as free (unesterified) plant sterol/stanol equivalents, rounded off at one decimal.

Pooled LDL-cholesterol effects were calculated while studies were categorised based on their PS dose (i.e. subgroup analysis with subgroups defined by the PS dose), using random-effects models according to the methods described by DerSimonian & Laird^(^ ^18^ ^)^. Random-effects models were used as they take into account the variation in LDL-cholesterol-lowering effects observed within and between studies. Studies were weighted by the inverse of their variance (1/se ^2^). Analyses were carried out for plant sterols and stanols combined and separately. When required, a more in-depth analysis was carried out to investigate the impact of food format on the LDL-cholesterol-lowering efficacy of PS. The pooled estimates and accompanying 95 % CI were determined using the PROC MIXED function of the SAS System (version 9.2; SAS Institute).

## Results

### Overview of the included studies

In total, 124 human studies with a total of 201 study arms were included in the present analysis. In 116 study arms, a parallel design was used whereas in eighty-five study arms, a cross-over design was used. Plant sterols and stanols were administered in 129 and fifty-nine study arms, respectively; in the remaining thirteen study arms, a mix of plant sterols and stanols was administered. The number of subjects per study arm was, on average, 48 (range 7–201). The average PS dose was 2·1 (range 0·2–9·0) g/d. In most of the studies, (low-fat) margarines/spreads or dairy-type products were used for enrichment with PS; other food formats included, among others, cereals, mayonnaise, salad dressing, soya products, bakery products, orange juice and vegetable oils. An overview of the included studies is given in online supplementary material.

### LDL-cholesterol-lowering effect of plant sterols and stanols combined and separately

**Fig. 1:**
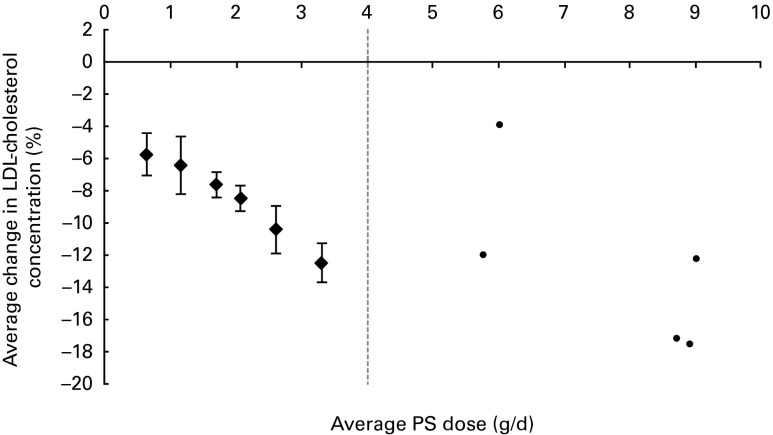
Average effects on LDL-cholesterol concentration for different dose ranges of phytosterols (PS) up to 4 g/d. The ● represent outcomes of single high-dose studies that were not pooled as these were scarce and scattered across a wide range of doses. Values are means, with 95 % CI represented by vertical bars.

**Fig. 2:**
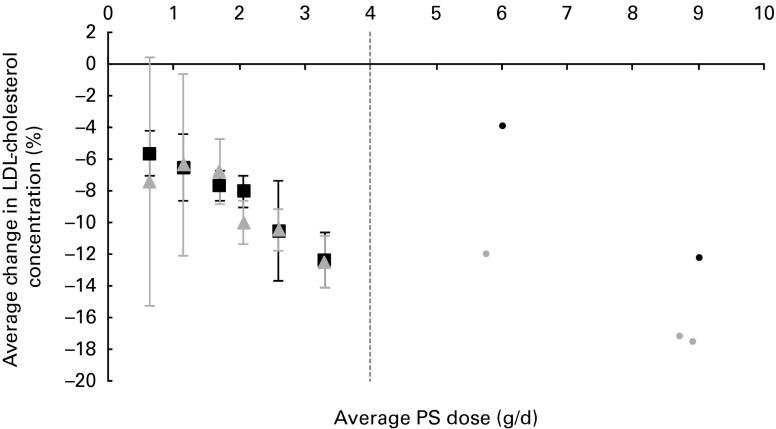
Average effects on LDL-cholesterol concentration for different dose ranges of phytosterols (PS), separately for plant sterols (■) and plant stanols (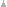). The ● represent outcomes of single high-dose studies that were not pooled as these were scarce and scattered across a wide range of doses. Values are means, with 95 % CI represented by vertical bars.

The average PS doses and relative effects on LDL-cholesterol concentrations for each of the defined dose ranges are summarised in Table 1. When plant sterols and stanols were analysed together, PS intakes were found to reduce LDL-cholesterol concentrations in a dose-dependent manner (*P*< 0·001; Fig. 1). When plant sterols and stanols were analysed separately, clear and comparable dose–response relationships were observed (Fig. 2). The impact of dose was significant in both analyses (*P*< 0·001 for plant sterols and *P*= 0·001 for plant stanols).

**Table 1:** Average LDL-cholesterol-lowering effect for different dose ranges of phytosterols (PS) combined and separately for plant sterols and stanols (Mean values and 95 % confidence intervals)

PS dose categories (g/d)*		Study arms (*n*)		Average PS dose (g/d)		Average LDL-cholesterol effect (%)
Combined	Plant sterols	Plant stanols
Mean	95 % CI	Mean	95 % CI	Mean	95 % CI
Dose < 1·0	24 (1 mix, 22 sterol, 1 stanol)	0·6	− 5·7	− 7·1, − 4·4	− 5·6	− 7·1, − 4·2	− 7·4	− 15·2, 0·4
≥ 1·0 dose < 1·5	13 (2 mix, 9 sterol, 2 stanol)	1·1	− 6·4	− 8·2, − 4·6	− 6·5	− 8·6, − 4·4	− 6·3	− 12·0, − 0·6
≥ 1·5 dose < 2·0	55 (7 mix, 39 sterol, 9 stanol)	1·7	− 7·6	− 8·4, − 6·8	− 7·6	− 8·6, − 6·7	− 6·7	− 8·8, − 4·7
≥ 2·0 dose < 2·5	60 (2 mix, 40 sterol, 18 stanol)	2·1	− 8·4	− 9·2, − 7·6	− 8·0	− 9·0, − 7·0	− 10·0	− 11·3, − 8·6
≥ 2·5 dose < 3·0	17 (0 mix, 6 sterol, 11 stanol)	2·6	− 10·3	− 11·8, − 8·9	− 10·5	− 13·7, − 7·3	− 10·4	− 11·7, − 9·1
≥ 3·0 dose ≤ 4·0	27 (1 mix, 11 sterol, 15 stanol)	3·3	− 12·4	− 13·6, − 11·2	− 12·3	− 14·0, − 10·6	− 12·5	− 14·1, − 10·8
*P* (dose effect)			< 0·001	< 0·001	0·001

* Studies carried out using doses exceeding 4 g/d were not included in the present analysis, as these were scarce and scattered across a wide range of doses; clustering them was judged to be inappropriate.

In the present analysis, in the dose category ≥ 2·0 dose < 2·5 g/d, an apparent difference of 2 % in LDL-cholesterol-lowering efficacy was observed between plant sterols and stanols. In *post hoc* analysis that was set up to investigate factors that might explain this finding, it was observed that the consistency of the food format (either solid/edible or liquid/drinkable) may play a role. In fact, within this particular dose category, fifteen of forty plant sterol studies used liquid food formats, whereas only four of eighteen stanol studies used this type of food format. Irrespective of the type of PS used, liquid foods lowered LDL-cholesterol concentrations by, on average, 6·5 %, whereas solid foods lowered LDL-cholesterol concentrations by, on average, 9·2 % (*P*= 0·003).

## Discussion

The present meta-analysis based on dose ranges showed that plant sterols and stanols lower LDL-cholesterol concentrations to a similar extent and in a dose-dependent manner, at least up to approximately 3 g/d. The observed comparability between plant sterols and stanols with regard to their cholesterol-lowering potential is in line with the findings of a recent meta-analysis^(^ ^16^ ^)^. In this meta-analysis^(^ ^16^ ^)^, fourteen studies that side by side compared the LDL-cholesterol-lowering efficacy of plant sterols with that of plant stanols at PS doses ranging from 0·6 to 3·3 g/d were included. Of the fifteen study arms reporting usable LDL-cholesterol data, seven study arms showed a non-significantly larger LDL-cholesterol-lowering effect for plant sterols than for plant stanols, whereas eight study arms showed a relatively larger effect for plant stanols than for plant sterols. Overall, it was concluded that plant sterols and stanols do not have statistically or clinically relevant differing effects on blood lipids. At higher intakes (>4 g/d), some individual studies suggest a larger LDL-cholesterol-lowering effect for plant stanols^(^ ^19^ ^,^ ^20^ ^)^ than for plant sterols^(^ ^21^ ^)^. However, high-dose studies are scarce and scattered across a wide range of PS doses (5·8–9·0 g/d). For proper high-dose equivalence testing, a direct comparison study would be needed with subjects on either high-dose plant sterol or high-dose plant stanol treatment being studied under the same conditions. As such a study has so far not been carried out, drawing conclusions on potential differences in efficacy between plant sterols and stanols at higher doses is not justified, as has been recently discussed by Plat *et al.* ^(^ ^13^ ^)^.

The dose dependency of the LDL-cholesterol-lowering effect of PS has previously been demonstrated in several meta-analyses^(^ ^6^ ^,^ ^9^ ^–^ ^12^ ^)^ and in individual dose–response studies^(^ ^19^ ^,^ ^22^ ^–^ ^24^ ^)^. So far, meta-analyses have suggested that the LDL-cholesterol-lowering effect of PS tapers off at intakes of 2–3 g/d with little additional benefit at higher intakes^(^ ^6^ ^,^ ^11^ ^)^. Consequently, several health authorities have included 2 g/d of PS from enriched foods as part of their diet and lifestyle guidelines in the management of hypercholesterolaemia^(^ ^7^ ^,^ ^8^ ^,^ ^25^ ^)^. From the present analysis, it appears that at least up to approximately 3 g/d of PS, there is a proportional dose–response effect. As the inhibition of cholesterol absorption by PS is probably a saturable process, some tapering-off effect would, however, be expected, but probably at doses slightly higher than 3 g/d. If indeed PS intakes >3 g/d lead to a greater LDL-cholesterol benefit, this would be meaningful from a clinical view point as additional LDL-cholesterol lowering could lead to a greater CVD risk reduction. However, the practical implications of higher PS intakes, such as the technical feasibility of incorporating higher amounts of PS into foods, cost–benefit aspects and, especially, the compliance of consumers, need to be considered. Based on research in populations that actually use foods with added PS, it appears that the intake of PS in real life is far below the recommendation^(^ ^26^ ^,^ ^27^ ^)^; on average, users consume 14 g/d of PS-enriched margarine, which corresponds to a PS intake of approximately 1 g/d. Therefore, encouraging people to consume PS at amounts exceeding approximately 3 g/d seems unrealistic. In addition, because of the observations of premature atherosclerosis in rare homozygous sitosterolaemic patients^(^ ^28^ ^)^ and due to epidemiological evidence suggesting a positive association between plasma plant sterol concentrations and CVD risk^(^ ^29^ ^)^, some concerns have been raised related to the increase in plasma plant sterol concentrations following high intakes of plant sterols from enriched foods. However, a recent meta-analysis summarised the totality of observational studies that investigated the association between modestly elevated plasma plant sterol concentrations and CVD risk and concluded that such an association does not exist^(^ ^30^ ^)^. Furthermore, plasma plant sterol concentrations after the intake of foods with added plant sterols remain below 1 % of total sterol concentrations circulating in the blood^(^ ^17^ ^)^. All in all, taking these aspects into account, the current recommendations to consume 2–3 g/d of PS for achieving a significant cholesterol-lowering effect seem to be still valid.

The use of different approaches to investigate dose–response relationships in meta-analyses may sometimes lead to different conclusions being drawn. For instance, Musa-Veloso *et al.* ^(^ ^12^ ^)^ previously concluded that the maximal LDL-cholesterol-lowering efficacy was greater for plant stanols (16·4 %) than for plant sterols (8·3 %) when analysing continuous dose–response curves. Also in the meta-analysis carried out by Demonty *et al.* ^(^ ^11^ ^)^, a non-significant 6·7 % difference in maximal cholesterol-lowering efficacy was observed between plant stanols and sterols based on continuous analysis. Such an approach offers the opportunity to predict the LDL-cholesterol-lowering effect of a given PS dose. However, the applied model seems to underestimate the LDL-cholesterol-lowering effect of plant sterols at doses of about 3 g/d. It is likely that this has affected the shape of the overall dose–response curve for plant sterols. This underestimation may have been caused by an unequal distribution of studies across the entire dose range. In fact, the availability of a large number of low-dose sterol studies with relatively high efficacy probably pulled the plant sterol curve towards a more curvy shape, whereas the stanol curve was mostly influenced by high-dose studies; indeed the number of stanol studies carried out using low doses ( < 1·5 g/d) was limited. The calculation of average effects for different dose ranges, as has been done in the present analysis, is less influenced by an imbalance of data points across the entire dose range. Moreover, this approach offers the opportunity to better take into account the large between-study variation by means of using random-effects models. On the other hand, one of the limitations of the dose–response approach is that the definition of the dose ranges is rather subjective. Especially between 1·5 and 2·5 g/d, small differences in cut-off values (e.g. < 2 or ≤ 2 g/d) could have a significant impact on the distribution of studies in the adjacent dose ranges and subsequently on the pooled averages for these particular dose ranges. In the present analysis, dose steps of 0·5 g/d were used between adjacent dose ranges, except for the outmost dose ranges, as these ranges would otherwise become too small. Although this approach led to a symmetrical distribution of the number of studies in the different dose ranges (*n* 24, *n* 13, *n* 55, *n* 60, *n* 17 and *n* 27 in ascending ranges), the ratio of plant sterol studies:plant stanol studies was disproportional by this definition (22:1, 9:2, 39:9, 40:18, 6:11 and 11:15, respectively). In any case, one should acknowledge that none of the dose–response approaches is ideal and should consider the pros and cons of the dose range *v.* the continuous approach before deciding which approach to choose for the research questions being addressed.

Besides the limitations of the applied dose–response method as discussed above, some other limitations should be mentioned. The present analysis was not set up as a typical meta-analysis, but in fact builds on previous published meta-analyses^(^ ^11^ ^,^ ^12^ ^)^ by highlighting the importance of using different analysis techniques. Therefore, heterogeneity tests and publication bias tests were not carried out. However, as between-study variation can never be ruled out, we decided beforehand to use random-effects models that take into account some of this variation. In addition, the baseline cholesterol concentration and the dose of PS have been shown to be important factors affecting the size of the LDL-cholesterol-lowering effect of PS^(^ ^6^ ^,^ ^10^ ^,^ ^11^ ^)^; by looking at relative changes and dose–response relationships, we believe that we have addressed these two important factors. Nevertheless, we cannot exclude that confounding by other factors, such as differences in food formats across the range of PS doses, might have affected the study outcomes. For example, in the present analysis, we found slightly lower efficacy for plant sterols than for plant stanols in the dose category ≥ 2·0 dose < 2·5 g/d; this was probably due to a larger number of liquid food formats among the plant sterol studies than among the plant stanol studies. PS in liquid foods *v.* solid foods might be less effective at lowering cholesterol concentrations due to a shorter transit time in the gastrointestinal tract. Also, liquid foods (drinks) are not per definition consumed together with a meal; sufficient ingestion of food (i.e. fat) is required to trigger bile release for PS to optimally compete with cholesterol for micellar incorporation and subsequently to optimally inhibit cholesterol absorption^(^ ^31^ ^)^. Given the substantial number of studies included, we assume that publication bias had not affected the findings severely. Lastly, the quality of studies was not assessed as we believe that rating study quality is a rather subjective exercise and it has not been shown that excluding low-quality studies leads to different conclusions^(^ ^10^ ^)^.

In summary, the present analysis showed that the LDL-cholesterol-lowering effect of PS continues to increase up to intakes of approximately 3 g/d to an average effect of 12 %. This was shown for both plant sterols and stanols. The importance of considering the advantages and disadvantages of different meta-analytical dose–response methods was discussed; future studies should decide on the most suitable dose–response approach depending on the research questions being addressed and the data available.

## Supplementary material

To view supplementary material for this article, please visit http://dx.doi.org/10.1017/S0007114514000750
